# Characteristics of burn injuries among children aged under six years in South Korea: Data from the Emergency Department-Based Injury In-Depth Surveillance, 2011-2016

**DOI:** 10.1371/journal.pone.0198195

**Published:** 2018-06-08

**Authors:** Joon Min Park, Yoo Seok Park, Incheol Park, Min Joung Kim, Kyung Hwan Kim, Junseok Park, Dong Wun Shin

**Affiliations:** 1 Department of Emergency Medicine, Inje University Ilsan Paik Hospital, Goyang, Korea; 2 Department of Emergency Medicine, Yonsei University College of Medicine, Seoul, Korea; Medical University Graz, AUSTRIA

## Abstract

Studies show that young children are vulnerable to burn injuries. We aimed to investigate the characteristics of thermal injuries in this population. We included children below 6 years of age who visited the emergency department (ED) after thermal injuries who were registered in the Korean Emergency Department-based Injury In-Depth Surveillance (2011–2016) database. Demographic characteristics, injury-related factors, and factors associated with ED treatment were gathered from the data. Then, we divided all children into two groups according to the ED discharge status: discharge versus admission (including cases transferred to other hospitals). The characteristics of the two groups were compared, and factors associated with admission were investigated. During the study period, 11,667 children with thermal injuries visited the ED. The number of boys was higher than the number of girls, and children aged 1 year accounted for the largest proportion. Most cases occurred in spring and indoors; the home was found to be the most common place. The most common type of burn was scald burns (69%), followed by contact burns (25.9%), and the most commonly burnt body area was the upper limbs (43.7%), followed by the lower limbs (16.8%). Most children (95.8%) were discharged home. The odds for hospital admission were lower for 2–3 and 4–5 year olds than for 0–1 year olds. The odds for hospital admission for contact burns were lower and those for electrical burns were higher than odds for hospital admission for scald burns. In summary, those aged 0–1 showed the largest incidence of thermal injuries and the most common burn mechanism was scald burns. Upper limbs were the most commonly affected body area, but their odds for requiring admission was lowest. Our results could be used as baseline data for prospective interventional studies investigating ways to reduce the incidence of childhood thermal injuries.

## Introduction

Burns are a major cause of injuries that result in death throughout the world. The annual number of fire-related deaths was estimated to be more than 0.3 million globally; however, this could be an underestimation if burn injuries from other sources such as hot materials and electrical current are not included [1]. Burn injuries can also cause permanent functional and cosmetic impairment, which might restrict interpersonal relationships and impede social life, and could thus be a burden not only to the victims and their families but also to the community. According to a report released by the World Health Organization, the annual loss of disability adjusted life years due to fire-related burn injuries is estimated to be as high as 10 million years [2].

Studies have identified children as the most vulnerable population to burn injuries, especially young children. Data from a single burn center in Korea reported that children aged <5 years accounted for the largest number of hospital admissions for burn injuries (26%) [3], which corroborates the trends observed in Iran (24.5%) and Nepal (39.7%) [4, 5]. Moreover, the mortality rates due to burn injuries are known to be the highest in young children. Data published by the World Health Organization in 2004 reported a global mortality rate from fire-related burn injuries of 3.9 per 0.1 million in children aged <20 years, 10.1 in infants ≤1 year of age, and 6.8 in those aged 1–4 years [6]. Although children are known to be a high-risk population for burn injuries, the causal factors and injury mechanisms are not well established. To this effect, several descriptive and case control studies were conducted throughout the world [6]. However, the documented factors largely varied because the inherent environment of each country was very different from each other.

In Korea, more than 500,000 patients (1,091 per 100,000 population) per year were treated for burn injuries during 2010–2014 [7]. In the same period, an average of 123,934 children (≤15 years) were treated annually, who accounted for 22.7% of all patients with burn injuries. Thus far, only a few studies have attempted to identify the epidemiologic factors of burn injuries in Korea. In particular, an emergency department (ED)-based or specific analysis of young children has not been conducted. This information would be helpful to comprehend the causal factors and establish preventive policies in the future. Therefore, we analyzed the data for burn injuries among a preschool population (age 0–5 years) using a nationwide multi-ED-based data registry for injuries in Korea.

## Methods

### Study design and data source

This retrospective observational study was conducted with the approval of the ethical review board of Inje University Ilsan Paik hospital, Goyang, Korea (IRB No: 2018-01-017-001). Due to the retrospective nature of our study, the requirement for informed consent was waived. The data used in this study were obtained from registers compiled by the Emergency Department based Injury In-Depth Surveillance (2011–2016), Korea Centers for Disease Control and Prevention. In addition, we obtained data that were fully anonymized before analysis. In this surveillance survey, the demographic and clinical information of patients who visited the ED for injuries was retrieved from their medical records by trained researchers from 23 institutions (S1 Appendix). The Korea Centers for Disease Control and Prevention offers continual education programs for researchers aimed at maintenance of the quality of data; all participating institutions undergo an assessment of their data quality and receive feedback periodically. Our study included pre-school children (<6 years) who developed thermal injuries due to heat including sunlight and electric current. Inhalation injuries were excluded in our analysis because selecting those with inhalation injuries from the registry data was not available.

### Data collection and categorization

The following variables were collected from the original data: sex, age, the month of ED visit, area where the injury occurred (indoors or outdoors), specific location where the injury occurred, transportation method, injury mechanism, principal diagnosis code, secondary diagnosis code, discharge from ED (home discharge, transfer to another hospital, hospital admission, and death), admission days, and the discharge from ward in the hospital admission cases. We defined season of ED visit as follows: winter (December to February), spring (March to May), summer (June to August), and autumn (September to November). The specific location where the injury occurred was categorized as home, commercial area, nature, educational institution, public area, and others. We categorized burn type in terms of the injury mechanism as follows: injuries from a hot liquid or steam (scald), hot solid (contact), flame or fire (flame), electric current (electric), and other natural or artificial sources of heat (others). Body area affected by the chief thermal injury was determined using the principal diagnostic code or, when not available, the secondary diagnosis code (head and neck, upper limbs, lower limbs, torso, and others [multiple sites, genitalia, or internal organs]. When the body area could not be determined from diagnostic codes, they were categorized as unspecified (S2 Appendix).

### Data analysis

The characteristics of all included cases were first analyzed, and then, the burn type and body area were assessed according to sex and age group for assessing the trends according to demographic factors. We categorized age into the three groups of 0–1 years (infant and early toddler), 2–3 years (toddler), 4–5 years (pre-school age). Then, we divided all patients into two groups according to the ED discharge status as follows: home discharge versus requiring hospital admission, and when the result was hospital admission, transfer to another hospital or death in the ED. The Chi-square test or Fisher’s exact probability test was used for the comparison of two groups. Data are expressed as the number with the percent for categorical variables, and as the mean with the standard deviation or the median and the interquartile range for numerical variables. Finally, a multiple logistic regression analysis was conducted to investigate the factors that might be associated with requiring hospital admission, and the variables that showed a difference between the two groups were used as confounders. All statistical analyses were conducted with SPSS statistics for Windows ver.21 (IBM, Armonk, NY, USA). A two-tailed p-value <0.05 was considered to indicate statistical significance.

## Results

### Overall characteristics of thermal injuries

**Fig 1:**
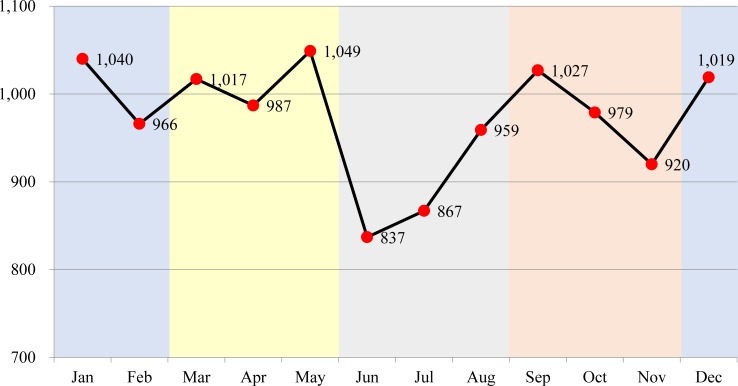
Month and season when the thermal injuries occurred.

Herein, a total of 11,667 children visited the hospital during the 6-year study period, and all of them were included in our analysis. The number of boys with thermal injuries was higher than the number of girls with thermal injuries; children aged 1 year accounted for the largest proportion of patients with thermal injuries. Spring and winter was the most common season during which the injuries occurred, and May, followed by January, was found to have the largest number of ED visits (Fig 1). Most cases occurred indoors, and the home was the most common location. The most common burn type was scald burns followed by contact burns, and the most common injured body area was the upper limbs followed by the lower limbs and torso. Most of the patients were discharged home (Table 1). For admitted cases, the median number of days of hospitalization was 7 days, with an interquartile range of 3–10 days. Of the admitted patients, 8 were transferred to another hospital and no cases of death were reported.

**Table 1:** Characteristics of thermal injuries reported during 2011–2016.

				N (%)	
**Sex**		Male		6550 (56.1)	
		Female		5117 (43.9)	
**Age (y)**		0		2360 (20.2)	
		1		4952 (42.4)	
		2		1904 (16.3)	
		3		1079 (9.2)	
		4		787 (6.7)	
		5		585 (5.0)	
**Season**		Winter		3025 (25.9)	
		Spring		3053 (26.2)	
		Summer		2663 (22.8)	
		Autumn		2926 (25.1)	
**Place (Environment)**		Indoors		11224 (96.2)	
		Outdoors		393 (3.4)	
		Unspecified		50 (0.4)	
**Specific location of event**		Home		10009 (85.8)	
		Commercial area		1077 (9.2)	
		Nature		120 (1.0)	
		Educational institution		105 (0.9)	
		Public area		101 (0.9)	
		Others		189 (1.6)	
		Unspecified		66 (0.6)	
**Transportation**		Self		10651 (91.3)	
		Ambulance		1012 (8.7)	
		Unspecified		4 (0)	
**Burn type**		Scald		8052(69.0)	
		Contact		3017 (25.9)	
		Flame		162 (1.4)	
		Electric		262 (2.2)	
		Others		174 (1.5)	
**Body area affected**		Upper limbs		5097 (43.7)	
		Lower limbs		1957 (16.8)	
		Head and neck		1299 (11.1)	
		Torso		1239 (10.6)	
		Others		112 (1.0)	
		Unspecified		1963 (16.8)	
**Discharge**		Home discharge		11177 (95.8)	
		Transfer to another hospital		326 (2.8)	
		Admission		163 (1.4)	
		Death		1 (0)	

### Burn type and injured body area according to sex and age

**Fig 2:**
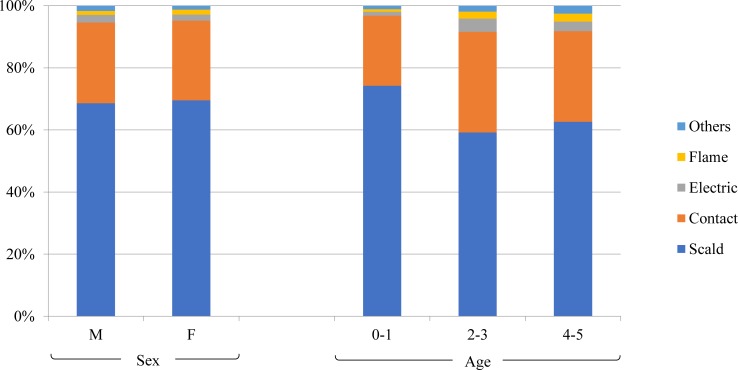
Type of thermal injuries according to sex and age group.

**Fig 3:**
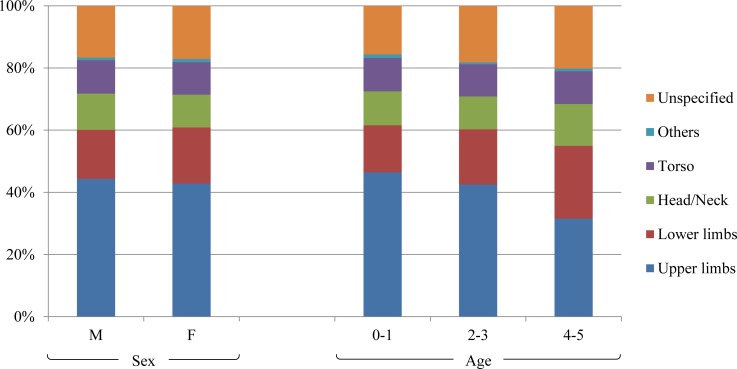
Affected body part according to sex and age group.

The most common burn type was scald burns in both sexes, and the proportion of each burn type in both sexes was similar (Fig 2). The most commonly injured body area was the upper limbs followed by the lower limbs and head/neck in both sexes (Fig 3). Across all ages, scald burns were the most commonly observed, but the proportion decreased in children older than 1 year (Fig 2). The proportion of contact and electric burns peaked in the 2–3-year-old age group. The upper limbs were the most commonly injured body part followed by the lower limbs in all age groups, but this showed a decreasing trend with age: 46.4% in 0–1 year olds, 42.5% in 2–3 year olds, and 31.6% in 4–5 year olds. On the other hand, the proportion of burned lower limbs was relatively high in the older age groups: 15.1% in 0–1 year olds, 17.8% in 2–3 year olds, and 23.4% in 4–5 year olds (Fig 3).

### Home discharge versus hospital admission

Sex, age group, transportation, burn type, and body area differed between the two groups, but the season and location where the injury occurred were not different (Table 2). The proportion of boys and children aged 0–1 was larger in the hospital admission group. The number of children who visited the ED via ambulance was higher in the hospital admission group. Although the proportion of scald and electric burns was larger in the hospital admission group, the proportion of contact burns was larger in the home discharge group. We also found that the proportion of affected head/neck and torso areas was larger in the hospital admission group, and the proportion of affected upper and lower limbs was larger in the home discharge group.

**Table 2:** Characteristics of thermal injuries in the discharge group and admission group.

		Discharge, N (%)	Admission, N (%)	P value
**Sex**	Boy	6253 (55.9)	297 (60.6)	0.04
	Girl	4924 (44.1)	193 (39.4)	
**Age group (y)**	0–1	6691 (62.3)	351 (71.6)	<0.01
	2–3	2890 (25.9)	93 (19.0)	
	4–5	1326 (11.9)	46 (9.4)	
**Season**	Winter	2908 (26.0)	117 (23.9)	0.51
	Spring	2921 (26.1)	132 (26.9)	
	Summer	2540 (22.7)	123 (25.1)	
	Autumn	2808 (25,1%)	118 (24.1)	
**Place (Environment)**	Indoor	10748 (96.2)	476 (97.1)	0.60
	Outdoor	379 (3.4)	14 (2.9)	
	Unspecified	50 (0.4)	0 (0)	
**Transportation**	Self	10304 (92.2)	347 (70.8)	<0.01
	Ambulance	869 (7.8)	143 (29.2)	
	Unspecified	4 (0)	0(0)	
**Burn type**	Scald	7627 (68.2)	425 (86.7)	<0.01
	Contact	2994 (26.8)	23 (4.7)	
	Flame	159 (1.4)	3 (0.6)	
	Electric	230 (2.1)	32 (6.5)	
	Other	167 (1.5)	7 (1.4)	
**Body area affected**	Upper limbs	4996 (44.7)	101 (20.6)	<0.01
	Lower limbs	1891 (16.9)	66 (13.5)	
	Head/Neck	1191 (10.7)	108 (22.0)	
	Torso	1114 (10.0)	125 (25.5)	
	Other	93 (0.8)	19 (3.9)	
	Unspecified	1892 (16.9)	71 (14.5)	

### Factors associated with hospital admission

Sex, age group, transportation method, burn type, and body area were finally included in a multiple logistic regression analysis (Table 3). Patients aged 2–3 and 4–5 years showed a low odds ratio (OR) for hospital admission when compared to those aged 0–1 years. Ambulance transportation demonstrated a high OR for admission compared to self-transportation. While contact burns showed a low OR for hospital admission compared to scald burns, electrical burns showed a high OR. Burns over the lower limbs, head/neck, torso, or other area showed higher ORs for hospital admission than burns over the upper limbs.

**Table 3:** Odds ratios of factors associated with admission.

		Unadjusted OR	95% CI	Adjusted OR	95% CI
**Sex**	Boy	Reference		Reference	
	Girl	0.83	0.69–0.99	0.83	0.67–1.02
**Age group (y)**	0–1	Reference		Reference	
	2–3	0.64	0.51–0.81	0.65	0.50–0.85
	4–5	0.69	0.50–0.94	0.63	0.43–0.90
**Transportation**	Self	Reference		Reference	
	Ambulance	4.89	3.97–6.01	3.02	2.38–3.84
**Burn type**	Scald	Reference		Reference	
	Contact	0.14	0.09–0.21	0.25	0.16–0.39
	Flame	0.34	0.11–1.07	0.14	0.02–1.03
	Electrical	2.50	1.70–3.66	5.75	3.35–9.88
	Other	0.75	0.35–1.61	0.54	0.16–1.75
**Body area affected**	Upper limbs	Reference		Reference	
	Lower limbs	1.73	1.26–2.37	1.61	1.16–2.23
	Head/Neck	4.49	3.40–5.93	4.15	3.10–5.56
	Torso	5.55	4.24–7.27	4.09	3.06–5.46
	Other	10.11	5.94–17.19	7.94	4.57–13.80

OR, odds ratio; CI, confidence interval

The level of significance validated with the Hosmer-Lemeshow test was 0.267.

## Discussion

Burns are a kind of injury that require complicated resources for care, such as sufficient medical equipment, well-trained staff, and a well-organized stepwise care system at the community or country level. For this reason, establishing preventive strategies is more rewarding than administering treatment [8]. Investigations on the epidemiology of burn injuries have important roles in that they can be used as baseline data for recognizing the present situation of burn injuries and can be further used for establishing preventive policies and burn care facilities [9]. Because the environment, demographics, and culture can differ, epidemiologic investigations of burns should be conducted in each country and linked with the resultant policies [10]. Considering that burn injury patients usually initially visit the ED, an ED-based investigation might be useful to confirm epidemiologic factors. Although some countries have conducted ED-based epidemiologic studies on burn injuries, they analyzed the data obtained at a single ED or did not analyze the pediatric population independently [10–13]. We investigated the characteristics of thermal injuries among children aged <6 years, a population that is known to be vulnerable to burns, using data drawn from a nationwide multi-ED (N = 23)-based injury registry.

Our study showed that boys visited the ED much more often than girls, especially in the hospital admission group, and this result is similar to that stated in previous studies conducted in other countries [8, 14, 15]. A possible explanation could be that boys have a more inquisitive nature and a more risk-taking behavior [6]. Younger children aged 0–1 years experienced thermal injuries far more often than those aged 4–5 years. This could be attributable to the fact that under 4 years of age, a child’s motor development usually does not keep up with the cognitive and intellectual development, and children are usually less likely to be injured by familiar indoor objects as they grow [6, 16].

Scald burns are known to be the most frequent type of burns among children aged under 6 years, typically by pulling down a container filled with hot fluid, in most geographic and economic groups [6]. Likewise, scald burns were the most common type of burns in both the discharge and admission group in our study. The proportion of scald burns in younger children aged 0–1 years was relatively higher than that in older children. Previous studies also reported that the proportion of burns other than scald burns increase in older children [17]. The second most common type was contact burns amongst all children, but electric burns in the admission group only. Only a small portion (0.8%) of children with contact burns required to be admitted, and from this, we can infer that contact burns are mostly mild. The regression analysis also indicated a lower odds for admission in children with contact burns when compared with children with scald burns (OR = 0.25).

The common types of burns (i.e. scald and electric burn) observed in the admission group were similar to those observed in previous retrospective cohort studies that investigated admitted patients in a burn center in Korea [3]. Besides scald burns, the second most common type of burns among admitted children differed across countries. The second most common type of burn amongst admitted children was flame burns in Iran and Turkey [8, 15] and contact burns in Australia [17]. We believe this difference indicates that the injury mechanism is affected by the living environment of each country.

The most commonly burnt body area was the upper limbs (43.7%) followed by the lower limbs (16.8%) in our study, and this is similar to trends observed in a previous study. In a nationwide epidemiologic study conducted in Taiwan, which investigated both inpatients and outpatients, the upper limbs were the most commonly affected area (46%), followed by the lower limbs (27%) among children aged 0–4 years [18]. However, the findings of our study indicated that the predominantly affected body area was the torso (25.5%) and head/neck (22.0%) in the admission group. Likewise, data from a single burn center in Taiwan regarding children with burns under the age of 15 years, showed that the most frequent injured body area was the trunk and head/neck [19]. Thus, burn injuries to limbs in children might be relatively minor than that occurring over other body areas, and therefore, admission is not usually warranted. The result of the regression analysis also supports this explanation.

A majority of childhood burns is known to occur at home [6]. Our results also indicate that most children were injured indoors, especially in the home (85.8%). Amongst children injured in the home, the kitchen/dining area (58.8%) was the most common area, followed by the living room (23.3%), bedroom (11.4%), bathroom (5.1%). Although ED-based burn injury data for children are not available worldwide, previous studies that investigated admitted children with burns reported similar outcomes. In the single burn center study conducted in Taiwan, most thermal injuries occurred in the home (93%), and in particular, in the kitchen/dining area (57.3%) and the living room (30.0%) [19]. In a study conducted in a single burn center in Iran, 89.7% of the children were injured in the home (58.2% in the kitchen and 17.5% in the living room) [8]. Flame sources for cooking, hot kitchen utensils, hot food and drinks might be the cause of burns occurring in the home. We believe that the incidence of childhood burns can be reduced through the development of safe kitchen devices and structural changes within residential spaces.

In our study, most children with thermal injuries visited the ED in spring and winter, and the least visited in summer. Considering that most of the thermal injuries occurred indoors, especially at home, the number of ED visits in summer, when outdoor activity is high, was relatively low. Owing to a lack of ED-based data for other countries, it is difficult to compare thermal injuries according to seasons with other countries. In the single burn center study conducted in Taiwan, children younger than 15 years were mostly admitted in winter (38.2%) and autumn (32.1%) [19]. On the other hand, a study conducted in a single burn center in Iran showed that children younger than 15 years of age were mostly admitted in summer (32.4%) [8]. Among children requiring admission in our study, no significant differences in ED visits were observed across the four seasons. The differences in age group (i.e. preschool age in our study vs. <15 years in other studies) and the living environment of each countries might be the chief cause of the differences in the peak seasonal distribution in the admission group.

This study has some limitations. First, because most data were retrieved from medical records, some data were not available. Although most of the missing data were minor, a large amount of data regarding burnt body areas (16.8%) were missing. However, the proportion of missing data was almost similar in both the home discharge and hospital admission group, and this could have minimized the impeding comparability between the two groups. Second, because our study analyzed data drawn from some of all EDs in South Korea, the possibility of selection bias is present. Third, some epidemiologic and clinical factors of thermal injury such as the presence of a caregiver and burn depth or size were not investigated in this study. In the future, prospective data collection including these factors is desirable to comprehend the characteristics of children with burns in more detail and to establish policies on preventing thermal injuries.

## Conclusions

In conclusion, our study investigated the characteristics of preschool children with thermal injuries who visited the EDs first in Korea. Burns occurred more in boys, and overall, the peak age for burns among all patients was approximately 1 year. An indoor environment, particularly the home, was the most common place that burns occurred. Scald burns were the most common burn type, and upper limbs were the most commonly affected body area. The results obtained from our study can be used as basic data for future studies investigating interventions for preventing thermal injuries in children. In addition, studies regarding factors proven to be related with the requirement for admission in particular might be required to reduce the incidence of severe thermal injuries in children.

## Supporting information

S1 Appendix (XLSX)

S2 Appendix (XLSX)
